# GMP-Grade Manufacturing of T Cells Engineered to Express a Defined γδTCR

**DOI:** 10.3389/fimmu.2018.01062

**Published:** 2018-05-30

**Authors:** Trudy Straetemans, Guido J. J. Kierkels, Ruud Doorn, Koen Jansen, Sabine Heijhuurs, Joao M. dos Santos, Anna D. D. van Muyden, Henri Vie, Béatrice Clemenceau, Reinier Raymakers, Moniek de Witte, Zsolt Sebestyén, Jürgen Kuball

**Affiliations:** ^1^Laboratory of Translational Immunology, University Medical Center Utrecht, Utrecht University, Utrecht, Netherlands; ^2^Department of Hematology, University Medical Center Utrecht, Utrecht, Netherlands; ^3^CRCINA, INSERM 1232, CNRS, Université d’Angers, Université de Nantes, Nantes, France; ^4^CHU de Nantes, Hôtel Dieu, UTCG, Nantes, France

**Keywords:** γδTCR, cancer immunotherapy, GMP-manufacturing, T cell engineering, cancer, TEG

## Abstract

γ9δ2T cells play a critical role in daily cancer immune surveillance by sensing cancer-mediated metabolic changes. However, a major limitation of the therapeutic application of γ9δ2T cells is their diversity and regulation through innate co-receptors. In order to overcome natural obstacles of γ9δ2T cells, we have developed the concept of T cells engineered to express a defined γδT cell receptor (TEGs). This next generation of chimeric antigen receptor engineered T (CAR-T) cells not only allows for targeting of hematological but also of solid tumors and, therefore, overcomes major limitations of many CAR-T and γδT cell strategies. Here, we report on the development of a robust manufacturing procedure of T cells engineered to express the high affinity Vγ9Vδ2T cell receptor (TCR) clone 5 (TEG001). We determined the best concentration of anti-CD3/CD28 activation and expansion beads, optimal virus titer, and cell density for retroviral transduction, and validated a Good Manufacturing Practice (GMP)-grade purification procedure by utilizing the CliniMACS system to deplete non- and poorly-engineered T cells. To the best of our knowledge, we have developed the very first GMP manufacturing procedure in which αβTCR depletion is used as a purification method, thereby delivering untouched clinical grade engineered immune cells. This enrichment method is applicable to any engineered T cell product with a reduced expression of endogenous αβTCRs. We report on release criteria and the stability of TEG001 drug substance and TEG001 drug product. The GMP-grade production procedure is now approved by Dutch authorities and allows TEG001 to be generated in cell numbers sufficient to treat patients within the approved clinical trial NTR6541. NTR6541 will investigate the safety and tolerability of TEG001 in patients with relapsed/refractory acute myeloid leukemia, high-risk myelodysplastic syndrome, and relapsed/refractory multiple myeloma.

## Introduction

Chimeric antigen receptor engineered T (CAR-T) cells are currently entering clinical practice with remarkable response rates resulting in multiple FDA approvals in 2017 (1). Major limitations of current clinical strategies are, however, that CAR-T cells rarely offer solutions to solid tumors. Another restriction of current CAR-T approaches is that target antigens are often present on healthy tissues. Therefore, we introduced the concept of metabolic cancer targeting through a defined high-affinity Vγ9Vδ2T cell receptor (TCR) (2) and proposed to utilize T cells engineered to express a defined γδT cell receptor (TEGs) as the next generation of CAR-T. Vγ9Vδ2TCRs sense spatial and conformational changes of butyrophilin 3A1 (CD277) and RhoB mediated by intracellular phosphoantigen accumulation (PAg). Transformed cells often have accumulated PAg due to a dysregulated mevalonate pathway, enabling γ9δ2T cells to recognize them (3, 4). The TEG concept allows for selecting the most potent Vγ9Vδ2TCR and targeting of liquid and solid tumors (5). TEGs also overcome the diversity of natural γ9δ2T cells (6) and avoid negative regulation of the Vγ9Vδ2TCR through innate receptors of γ9δ2T cells (7). In addition, as Vγ9Vδ2TCR are introduced in both CD8 effector and CD4 helper cells, TEGs can deliver professional help through, e.g., maturing dendritic cells (5). For clinical testing of the TEG concept, we recently selected a highly tumor reactive Vγ9Vδ2TCR clone (clone 5) from the natural repertoire of a healthy individual (2). This particular Vγ9Vδ2TCR showed a strong reactivity toward a broad range of tumor cells within the TEG format, including primary leukemic blasts (8) as well as primary multiple myeloma cells (9). Due to the selection of a high-affinity Vγ9Vδ2TCR, TEGs also outperform natural γ9δ2T cells in terms of direct tumor recognition (2). For administration of TEGs in human, we recently proposed a purification step of TEGs by depletion of non- and poorly-engineered cells in order to further increase activity and definition of the product (8). However, a Good Manufacturing Practice (GMP)-grade procedure for a TEG drug product has not yet been defined. In this article, we describe the developmental process from a “research method” (8) to a manufacturing procedure that is fully compliant with GMP. Given that this process requires connecting two completely different worlds, a flexible research environment with a rigid GMP environment, the reported developmental process can be of high interest to researchers who aim at translating research findings to the clinic.

## Materials and Methods

### Production of Master Cell Bank (MCB) and Viral Vector Stock

The retroviral vector supernatant was produced in 293Vec-RD114 cells, a 293SF-based packaging cell clone producing RD114 pseudotyped viral particles containing MP71:TCRγ5-T2A-TCRδ5 transgene cassette, by BioNTech (Idar-Oberstein, Germany) (10, 11). To establish this packaging clone, first, a primary seed clone was established in a two-step transfection–transduction protocol. Candidate monoclonal cells were tested for the presence of the TCR transgene using qPCR. Transgene-positive clones were expanded to 14 cm Petri dishes in order to harvest supernatant. Primary seed clones were screened for virus titer production and the most productive cell clone (the producer cell line) was selected to grow a MCB. The MCB was released according to predefined criteria and stored in liquid nitrogen. Sequence integrity of the transgene was confirmed by sequence analysis of the MCB and TEG001 drug product samples.

### Preparation of Leukapheresis Material

Patient-derived mononuclear cells obtained by leukapheresis were cryopreserved in freezing medium [sodium chloride (NaCl) 0.9% with 10% dimethylsulfoxide and 5% human albumin (HA)]. The material was thawed at 37°C and mixed with five volumes of leukapheresis thaw medium [X-VIVO 15 chemically defined medium without gentamicin and phenol red (Lonza, Breda, The Netherlands), hereafter, called X-VIVO 15, supplemented with 10% HA]. After washing, cells were resuspended in culture medium with cytokines (X-VIVO 15 medium with 5% human serum), 1.7 × 10^3^ IU/ml of MACS GMP Recombinant Human interleukin (IL)-7 (Miltenyi Biotec, Bergisch Gladbach, Germany), and 1.5 × 10^2^ IU/ml MACS GMP Recombinant Human IL-15 (Miltenyi Biotec).

### Activation of T Cells

The cell suspension was diluted to a concentration of 1 × 10^6^ T cells/ml with culture medium containing cytokines. T cells were activated by adding anti-CD3/CD28 Dynabeads (Thermo Fisher Scientific, Etten-Leur, the Netherlands) to the cell suspension at a bead to cell ratio of 1:5 or otherwise indicated, homogenizing for 30 min at room temperature (RT) under rocking conditions, and subsequently incubating for 40–50 h at 37°C/5% CO_2_.

At Day 2, activated T cells were harvested by centrifugation and subsequently resuspended in culture medium containing cytokines. Manual cell count using trypan blue exclusion was performed and the cell suspension was further diluted with culture medium containing cytokines to a target concentration of 0.5 × 10^6^ viable cells/ml.

### Transduction

Non-tissue culture treated 24-well plates (Thermo Fisher Scientific) were coated with Retronectin (Takara Bio, Saint-Germain-en-Laye, France) at saturating conditions and incubated for 40–50 h at 2–8°C. At the day of transduction, the coated plates were incubated for 30 min at 37°C with 0.4% HA in NaCl, 0.9% to block unspecific binding. Next, viral supernatant was thawed at RT and diluted 1:1 with X-VIVO 15 medium, or as described in the relevant figure. RetroNectin-coated plates were coated with 2.0 ml/well diluted viral supernatant by spinning for 90 min at 500 × *g* at RT (one-spin hit transduction). The remaining supernatant was aspirated and discarded. Subsequently, 1 × 10^6^ activated cells were added per well of the viral-supernatant-coated plates (2.0 ml cell suspension of 0.5 × 10^6^ cells/ml) and incubated for 16–24 h at 37°C/5% CO_2_.

At Day 3, transduced cells were harvested from the 24-well plate, centrifuged, and subsequently resuspended in culture medium with cytokines. Manual cell count was performed and the cell suspension was further diluted with culture medium with cytokines to a final target concentration of 0.25 × 10^6^ viable cells/ml. The cell suspension was transferred to MACS GMP Cell Differentiation Bag(s) (Miltenyi Biotec) and incubated for 60–80 h at 37°C/5% CO_2_.

### Expansion of Transduced Cells

Transduced cells were cultured from Day 3 to Day 13. At Day 6, samples from cell suspension were taken to determine the concentration of viable cells by trypan blue exclusion. Transduction efficiency was determined by flow cytometry (% γδTCR positive T cells). The cell suspension was centrifuged and cultured in fresh culture medium supplemented with cytokines to a target concentration of 0.25 × 10^6^ viable cells/ml and incubated for 36–48 h at 37°C/5% CO_2_.

At Day 8, manual cell count was performed to determine the concentration of viable cells by trypan blue exclusion. The cell suspension, if applicable, was diluted to a target viable cell concentration of 1 × 10^6^ cells/ml with fresh culture medium without cytokines. The total volume of cell suspension was then supplemented with half the cytokine concentration. The cell suspension was incubated for 36–48 h at 37°C/5% CO_2_.

At Day 10, manual cell count was performed to determine the concentration of viable cells by trypan blue exclusion. The cell suspension was centrifuged and further diluted with fresh culture medium supplemented with cytokines to a target viable cell concentration of about 1 × 10^6^ cells/ml. The cell suspension was incubated for 60–80 h at 37°C/5% CO_2_.

### Purification of TEG001 by Research MACS Depletion of non- and poorly-engineered Immune Cells

pMP71: γTCR-T2A-δTCR-transduced T cells were incubated with biotin-labeled anti-αβTCR antibody (clone BW242/412; Miltenyi Biotec), followed by incubation with an anti-biotin antibody coupled to magnetic beads (anti-biotin MicroBeads; Miltenyi Biotec). Next, the cell suspension was applied to an LD column in a QuadroMACS™ Separator. αβTCR-positive T cells were depleted by MACS cell separation according to the manufacturer’s protocol (Miltenyi Biotec).

### Purification of TEG001 by CliniMACS Depletion of Non- and Poorly-Engineered Immune Cells

At Day 13, the cell suspension volume was reduced, when necessary, to 150–200 ml by removing supernatant after centrifugation. Anti-CD3/CD28 beads were removed from the cell suspension of transduced T cells using a magnet (Dynamag Cell Therapy Systems magnet).

The cell suspension was processed as follows:

a) Washed with phosphate buffered saline/ethylenediaminetetraacetic Acid/HA buffer (PBS/EDTA buffer with 0.5% HA) and adjusted to a volume of 95 ml with PBS/EDTA/HA buffer.
b) Incubated with 7.5 ml of TCRαβ-Biotin reagent (biotin-labeled anti αβTCR antibody (clone BW242/412; Miltenyi Biotec)) for 30 min on a swivel plate.
c) Washed with 600 ml PBS/EDTA/HA buffer and after centrifugation, the volume was adjusted to 190 ml with PBS/EDTA/HA buffer.
d) Incubated with 15 ml of anti-Biotin reagent (anti biotin antibody coupled to magnetic beads) for 30 min on a swivel plate.
e) Washed by adding PBS/EDTA/HA buffer to a volume of about 600 ml and removing supernatant after centrifugation. Subsequently, PBS/EDTA/HA buffer was added to a volume of about 200 ml and the αβTCR-expressing T cells (non- and poorly-engineered cells) were depleted using a CliniMACS Plus instrument (Magnetic Activated Cell Sorting) cell separation, program “depletion 3.1.”
f) Washed twice with infusion medium (NaCl 0.9% for infusion with 4% HA) and resuspended in infusion medium to obtain 25 ml TEG001 drug substance.

### Cells and Cell Lines

Daudi (CCL-213) was obtained from the American Type Culture Collection and ML-1 was obtained from Sigma-Aldrich (Zwijndrecht, the Netherlands). Cell lines were authenticated by short tandem repeat profiling/karyotyping/isoenzyme analysis. All cells were passaged for a maximum of 2 months, after which new seed stocks were thawed for experimental use. In addition, all cell lines were routinely verified by growth rate, morphology, and/or flow cytometry and tested negative for mycoplasma using MycoAlert Mycoplasma Kit. Daudi and ML-1 were cultured in RPMI + 1% Pen/Strep + 10% FCS (Bodinco, Alkmaar, the Netherlands). Peripheral blood mononuclear cells (PBMCs) were isolated from buffy coats or apheresis material obtained from the Sanquin Blood Bank (Amsterdam, the Netherlands).

### Flow Cytometry

Antibodies used for flow cytometry include: pan-γδTCR-PE (clone IMMU510; Beckman Coulter, Woerden, the Netherlands), pan-αβTCR-APC (clone IP26; eBioscience, Thermo Fisher Scientific), CD4-V450 (clone RPA-T4; BD Biosciences), CD8α-PerCP-Cy5.5 (RPA-T8; Biolegend), CD3-eFluor 450 (OKT-3; eBioscience), CD45-FITC (2D1; BD Biosciences), CD16-FITC (3G8; BD Biosciences), CD56-FITC (MY31; BD Biosciences), CD27-APC-eFluor780 (O323; eBioscience), CD45RO-PE-Cy7 (UCHL-1; BD Biosciences). All samples were analyzed on a BD LSRFortessa using FACSdiva software (BD Biosciences).

### ELISPOT and ELISA Assays

IFNγ ELISPOT was performed as previously described (5, 6). Briefly, 15,000 TCR-transduced or mock-transduced (TEG-LM1) T cells and 50,000 target cells (ratio 0.3:1) were cocultured for 24 h in nitrocellulose-bottomed 96-well plates (Merck, Schiphol-Rijk, the Netherlands), pre-coated with anti-IFNγ antibody (clone 1-D1K) (Mabtech, Nacka Strand, Sweden). Plates were washed and incubated with a second biotinylated anti-IFNγ antibody (clone 7-B6-1) (Mabtech) followed by streptavidin-HRP (Mabtech). IFNγ spots were visualized with tetramethylbenzidine substrate (Sanquin) and the number of spots was quantified using ELISPOT Analysis Software (Aelvis, Hannover, Germany). IFNγ ELISA was performed using ELISA-ready-go! Kit (eBioscience) following manufacturer’s instructions. Effector and target cells (E:T 15,000:15,000) were incubated for 24 h in the presence of pamidronate when indicated.

### Statistical Analyses

Differences were analyzed using indicated statistical tests in GraphPad Prism 7 for Windows (GraphPad Software Inc., La Jolla, CA, USA).

## Results

### Defining Optimal Activation of Primary T Cells With Anti-CD3/CD28 Coated Beads

**Figure 1:**
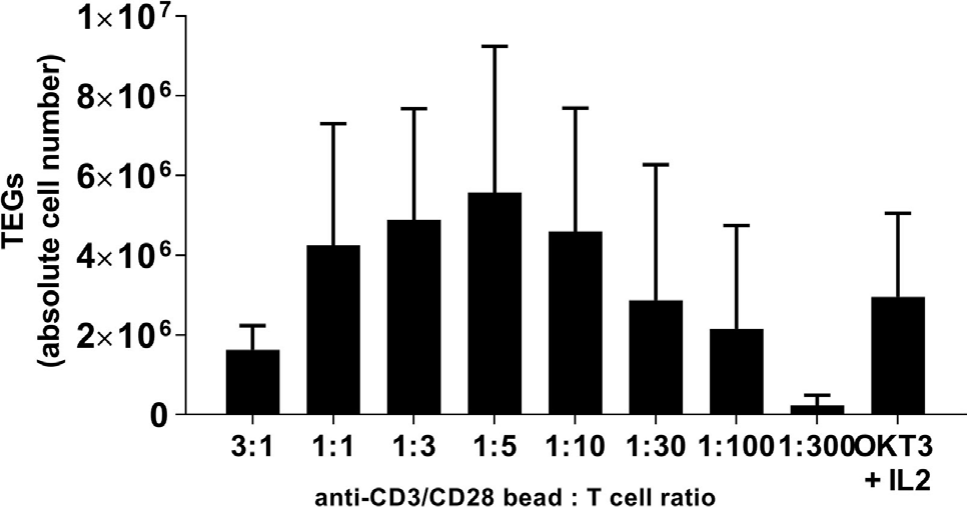
TEG yield depends on the optimal anti-CD3/CD28 bead to T cell ratio at the day of T cell activation. Multiple anti-CD3/CD28 bead to T cell ratios were tested and compared with respect to total TEG yield at day 10. After activation, transduction and expansion TEG numbers were defined by combining viable cell count with flow cytometry for γδTCR+ T cell percentage. OKT3 + IL2 served as a control activation and expansion stimulus. Mean absolute cell number + SD is shown, *n* = 2–3. The differences according to Mann–Whitney *U* tests are not significant (*p* > 0.05).

Stimulation of T cells with immobilized anti-CD3 and anti-CD28 antibodies provides both an antigen stimulus and co-stimulation for optimal T cell activation and expansion (12). Consequently, anti-CD3/CD28-coated beads are widely applied to engineer cellular products for different types of adoptive αβT cell therapies (13–16). The optimal anti-CD3/28 bead to CD3+ T cell ratio for activation, transduction with viral supernatant, and expansion of engineered immune cells is, however, frequently dependent on the specific transgene and production process. In order to define the best anti-CD3/28 bead to CD3+ T cell number for engineering TEGs, PBMCs were first analyzed for CD3+ T cell content by flow cytometry, and then incubated with various ratios of anti-CD3/CD28 beads in the presence of the cytokines interleukin (IL)-7 and IL-15. As a control stimulus, soluble OKT-3 and IL-2 was used. Next, T cells were transduced with non-GMP grade retroviral supernatant and expanded as described in the Section “Materials and Methods.” After 10 days, the total number of TEGs, defined as double positive TEGs when expressing γδTCRs and αβTCRs or single positive TEGs when expressing γδTCRs only, were assessed by flow cytometry. The mean total cell number of single and double positive TEGs ranged from 2 to 6 × 10^6^ cells when stimulated with anti-CD3/28 beads, and peaked at an anti-CD3/28 bead to T cell ratio of 1:5, while our standard OKT3 research protocol delivered 3 × 10^6^ cells (Figure 1). Due to the limited number of replicates, when performing a Mann–Whitney *U*-test, the difference between none of the conditions was significant (*p* > 0.05). A 1:5 bead to T cell ratio was chosen for the activation of T cells in all following TEG manufacturing procedures. This ratio is sufficient to activate T cells and is substantially lower in numbers than advised by the manufacturer and, therefore, saves costs during the future production procedures.

### Selection of a GMP-Grade Retroviral Producer Cell Clone

**Figure 2:**
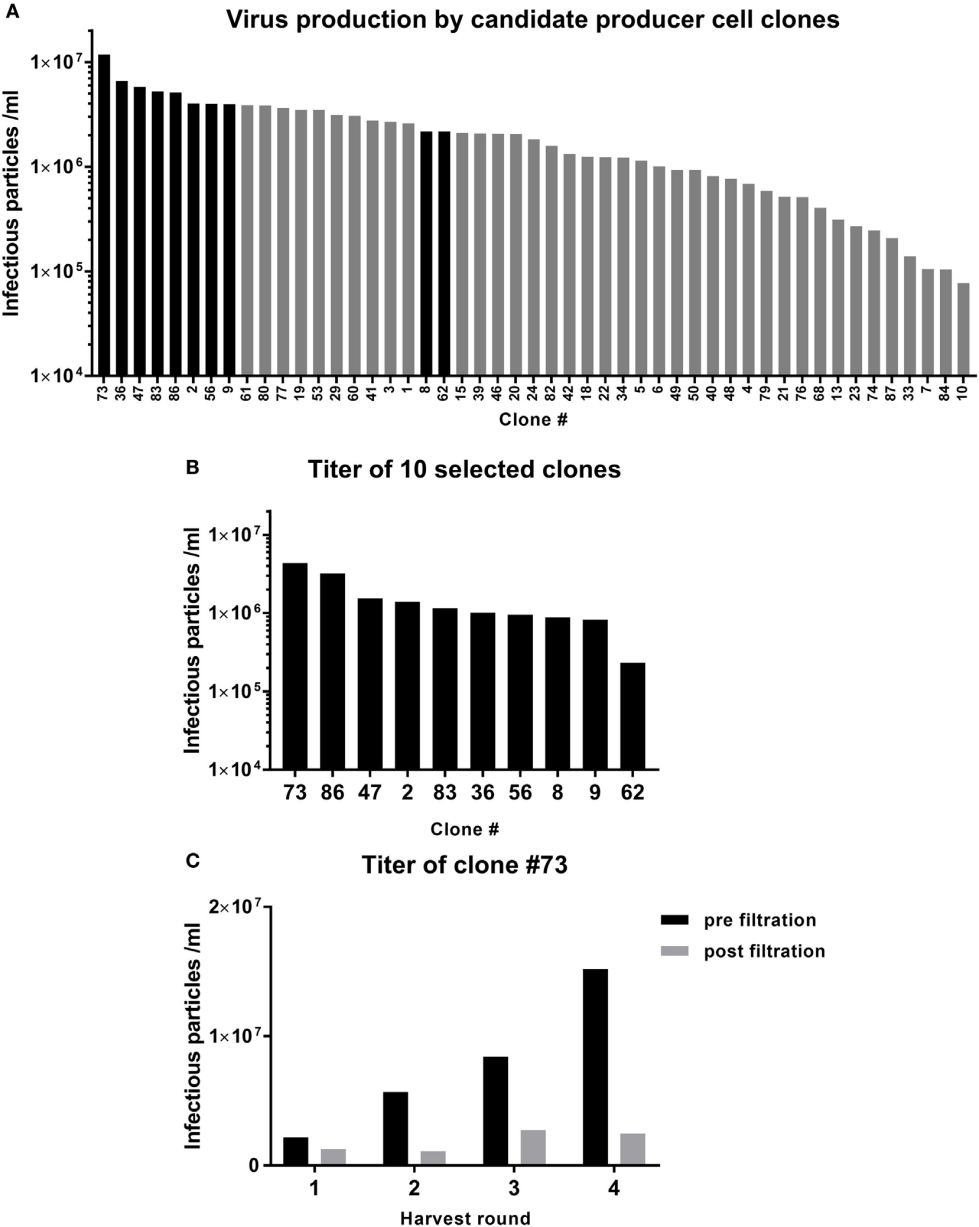
Selection of a viral vector producer cell clone. Retroviral supernatant was produced in 293vec-RD114 packaging cells. **(A)** The titer produced by the different clones was assessed in Jurkat cells. The clones depicted by the black bars were selected for a second round of testing **(B)**. **(C)** Clone #73 was picked for production of the TEG001 Good Manufacturing Practice retroviral supernatant. The titer was assessed after four rounds of harvest, before, and after filtration.

One of the most critical raw materials of the TEG manufacturing process is the viral vector supernatant used to transduce the T cells. Selection of a potent GMP-grade cell clone that produces the retroviral vector encoding the γδTCR is, therefore, critical for the success of the GMP-grade transduction process. The retroviral vector supernatant was produced using 293SF-based packaging cells, 293Vec-RD114, by BioNTech (10, 11). A large number of engineered producer cell clones were generated and the virus titer in the supernatant was assessed by titration experiments on Jurkat cells and analysis of γδTCR positive cells by flow cytometry (Figure 2A). The 8 best clones were selected for the second round of testing. Two additional clones from the upper midfield (#8, #62) were added to confirm the ranking (Figure 2B). Clone #73 was selected as the best GMP-grade retroviral producer cell line and was, therefore, further expanded and the titer was assessed before and after 0.45 µm filtration of the supernatant from different harvesting runs (Figure 2C). Filtration was performed in order to eliminate cell debris, a key step for generating GMP grade viral supernatant. This associated, however, with an up to sixfold reduction in viral particles in different harvesting runs (Figure 2C).

### Virus Titer Impacts Transduction Efficiency of Primary T Cells

**Figure 3:**
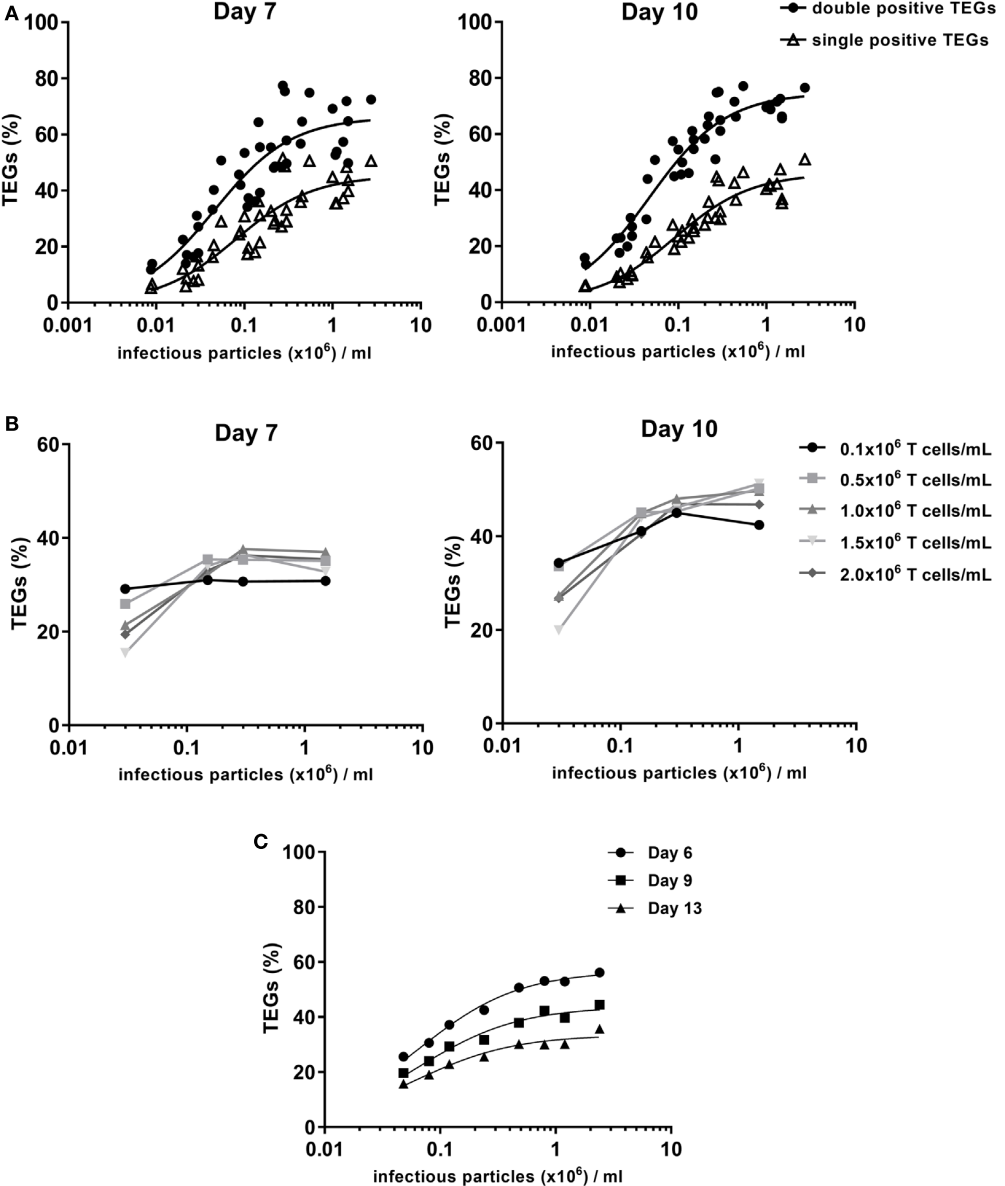
Transduction efficiency depends on the virus titer. T cells were activated with 1:5 CD3/CD28 bead to cell ratio followed by retroviral transduction with the γδTCR and expansion. **(A)** 0.5 × 10^6^ cells per ml were transduced with different concentrations of pre-Good Manufacturing Practice (GMP) viral supernatant. The % TEGs are shown as total double positive TEGs (black circles) and single positive TEGs (open triangles) at day 7 and day 10 after activation. **(B)** The relationship between T cell concentration and transduction efficiency was investigated. Transduction efficiency was evaluated after a 7- and 10-day expansion period, for a range of T cell concentrations during transduction and four different virus titers. **(C)** 0.5 × 10^6^ cells/ml were transduced with different concentrations of GMP viral supernatant to determine the relationship between virus titer and transduction efficiency after a 6-, 9-, and 13-day expansion period.

In the manufacturing of genetically modified cellular medicines, there is a strong relationship between transduction efficiency and the ability to produce sufficient cell numbers that meet predefined quality criteria (17). To optimize the production process we, therefore, assessed the amount of virus needed for optimal efficiency in a one-hit transduction. Viral supernatant generated in different pre-GMP proof runs from producer cell line clone #73 was used with virus titers ranging from 8.7 × 10^3^ to 2.7 × 10^6^ infectious particles (ip) per milliliters. Transduction efficiency was evaluated after 7 and 10 days of expansion with the optimized 1:5 anti-CD3/28 bead to T cell ratio. The percentage of TEGs was determined by flow cytometry using a pan-γδTCR antibody. The total number of both single and double positive TEGs was determined. Viral supernatant containing 1 × 10^6^ ip/ml provided transduction efficiencies of 60–70% TEGs (Figure 3A). The majority of TEGs showed high expression levels of γδTCR while being negative for αβTCR due to the successful competition of the introduced γδTCR chains against endogenous αβTCRs for components of the CD3 complex as reported (8). Thus, virus titers around 1 × 10^6^ ip/ml are sufficient for the generation of TEGs.

### Impact of T Cell Density on Transduction and Expansion Efficiency

It has been suggested that the density of activated T cells during the transduction procedure influences the transduction efficiency (18). Therefore, the optimal cell concentration during transduction was investigated within the context of four different virus titers (range 0.03–1.5 × 10^6^ ip/ml), and five different T cell densities (range 0.1–2.0 × 10^6^/ml). During the expansion phase, T cell densities were adjusted to defined concentrations at day 3 and 6 (both 0.25 × 10^6^/ml), and day 8 and 10 (both 1.0 × 10^6^/ml). The percentage of γδTCR-positive T cells was determined after 7 and 10 days by flow cytometry using a pan-γδTCR antibody (Figure 3B). Differences in TEG transduction efficiencies were only observed for very low virus titer conditions (0.03 × 10^6^ ip/ml). A cell concentration of 0.5 × 10^6^/ml was selected as the standard cell density during transduction for TEG001 manufacturing process.

### Impact of GMP-Grade Virus Titer From Clone #73 on Transduction Efficiency

Due to procedural differences between the manufacturing of research-grade and GMP-grade retroviral supernatant, the relationship between virus titer and transduction efficiency for the final GMP viral supernatant batch, which will be used for the production of TEGs for the clinical trial, was further investigated. T cells were transduced at a density of 0.5 × 10^6^ cells/ml with different dilutions of GMP-grade viral supernatant in a one-spin hit transduction procedure. The transduction efficiency and total γδTCR positive cell numbers were evaluated after 6, 9, and 13 days by flow cytometry using a pan-γδTCR antibody (Figure 3C). The percentage of TEGs with the different pre-GMP titers was highest between day 6 (Figure 3C) and day 10 (data not shown) followed by a small decrease, most likely in line with our previous observation that engineered immune cells have a slight disadvantage in proliferative capacity early after transduction when compared to non engineered immune cells (19). In line with the observation for pre-GMP-grade viral supernatant, the final GMP-grade viral supernatant that will be used for the clinical study provided transduction efficiencies of up to 60% TEGs when utilizing a titer of 1.2 × 10^6^ ip/ml (Figure 3C).

### Higher γδTCR Expression Increases Antitumor Activity

**Figure 4:**
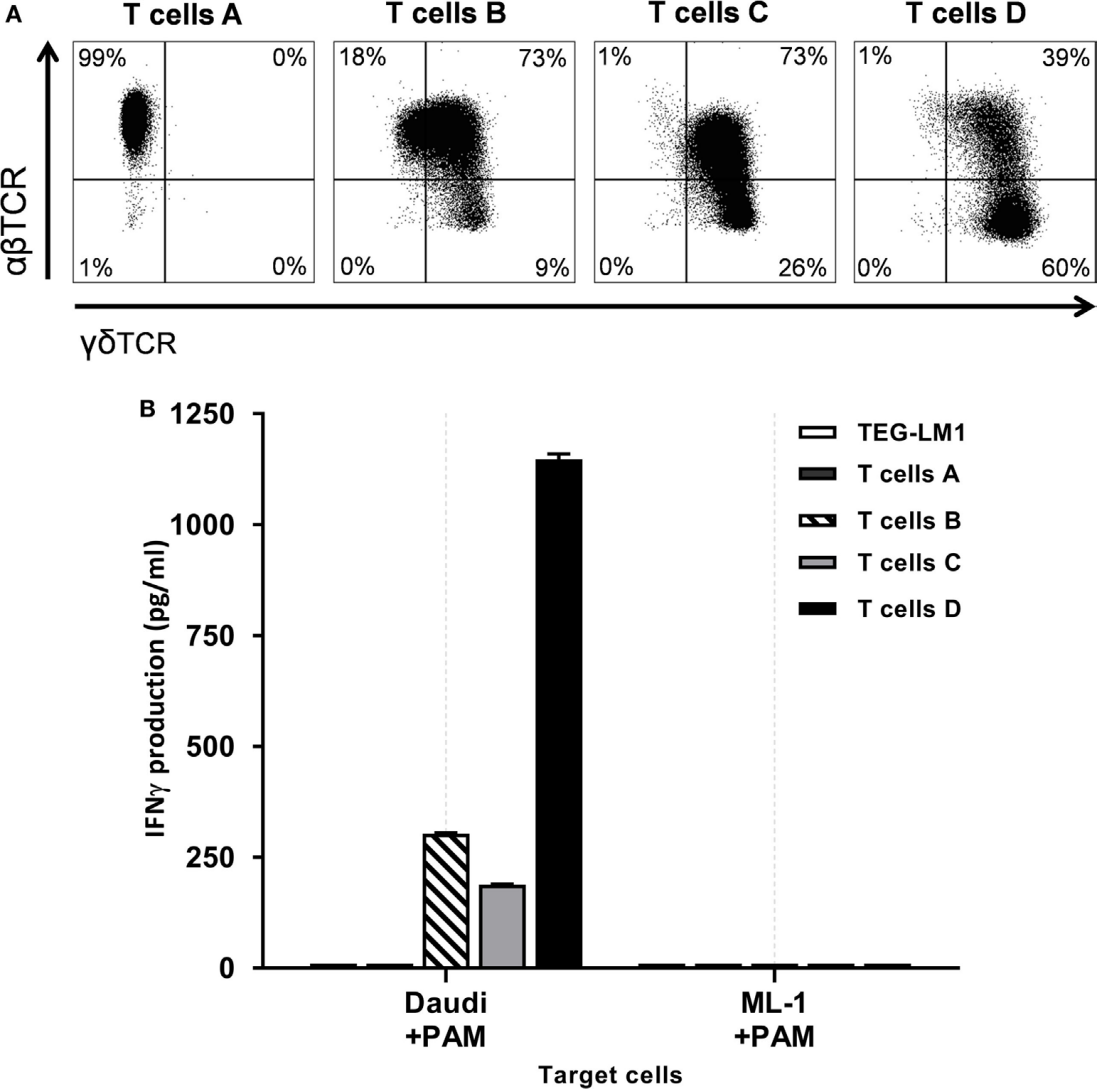
γδTCR expression defines functional activity. **(A)** A defined CD4+ T cell clone (20) underwent the transduction procedure but remained untransduced (T cells A) or was transduced with the MP71:TCRγ5-T2A-TCRδ5 retroviral vector, resulting in low and intermediate γδTCR single positive cell lines (T cells B and C, respectively). Primary T cells were used to generate the cell line with a high single positive γδTCR fraction that was further purified for CD4+ T cells by CD4 MACS selection after transduction (T cells D). In all cases, after one cycle of expansion, γδTCR and αβTCR expression was measured in the viable CD45+ gate by flow cytometry after which the cells were used in a function assay. **(B)** Different T cells were coincubated with the indicated tumor target cell lines in the presence of pamidronate in triplicate. Daudi is the prototypic TEG001 positive target, ML-1 is the negative control target. TEG-LM1 served as the negative control effector. After 20 h at 37°C, supernatant was harvested and analyzed by IFNγ ELISA. Mean IFNγ production + SD is shown.

Defining a potency assay for a medicinal product is critical for assessing whether the final product is biologically active. Previous reports suggested that γδTCR expression levels correlate with activity of TEGs (5, 8). To formally confirm that indeed γδTCR expression is key for TEG activity, we used a defined CD4+ T cell clone (20), which underwent the transduction procedure but remained untransduced (T cells A) or was transduced with the MP71:TCRγ5-T2A-TCRδ5 retroviral vector, resulting in low and intermediate γδTCR single positive cell lines (T cells B and C, respectively). Primary T cells from a GMP proof run were used to generate the cell line with a high single positive γδTCR fraction that was further purified for CD4+ T cells by CD4 MACS selection after transduction (T cells D) (Figure 4A). Next, activity of theses γδTCR cell lines with different amounts of single γδTCR-positive T cells was compared side by side in the presence of pamidronate against Daudi as a positive, or ML1 as a negative tumor target (Figure 4B). CD4+ TEGs with higher γδTCR expression had a higher activity in terms of IFNγ cytokine secretion when compared to CD4+ T cell clones with lower or literally absent γδTCR expression. As control, T cells A–C, which express an endogenous allogeneic HLA-DPB1*04:01-reactive αβTCR, were coincubated with an HLA-DPB1*04:01 expressing B cell line. This resulted in cytokine levels equivalent or higher than from T cells D, indicating that T cells A–C were highly functional when triggered by the endogenous TCR (data not shown). These data are in line with previous reports from our group (8) and support the rationale to enrich in the GMP process only for TEGs with highest γδTCR expression. Therefore, we defined γδTCR-positive expression as a potency assay for TEGs and γδTCR single positive TEGs defines the functionally most active population.

### Enrichment of TEGs by CliniMACS Through Depletion of Non- and Poorly-Engineered Immune Cells

**Figure 5:**
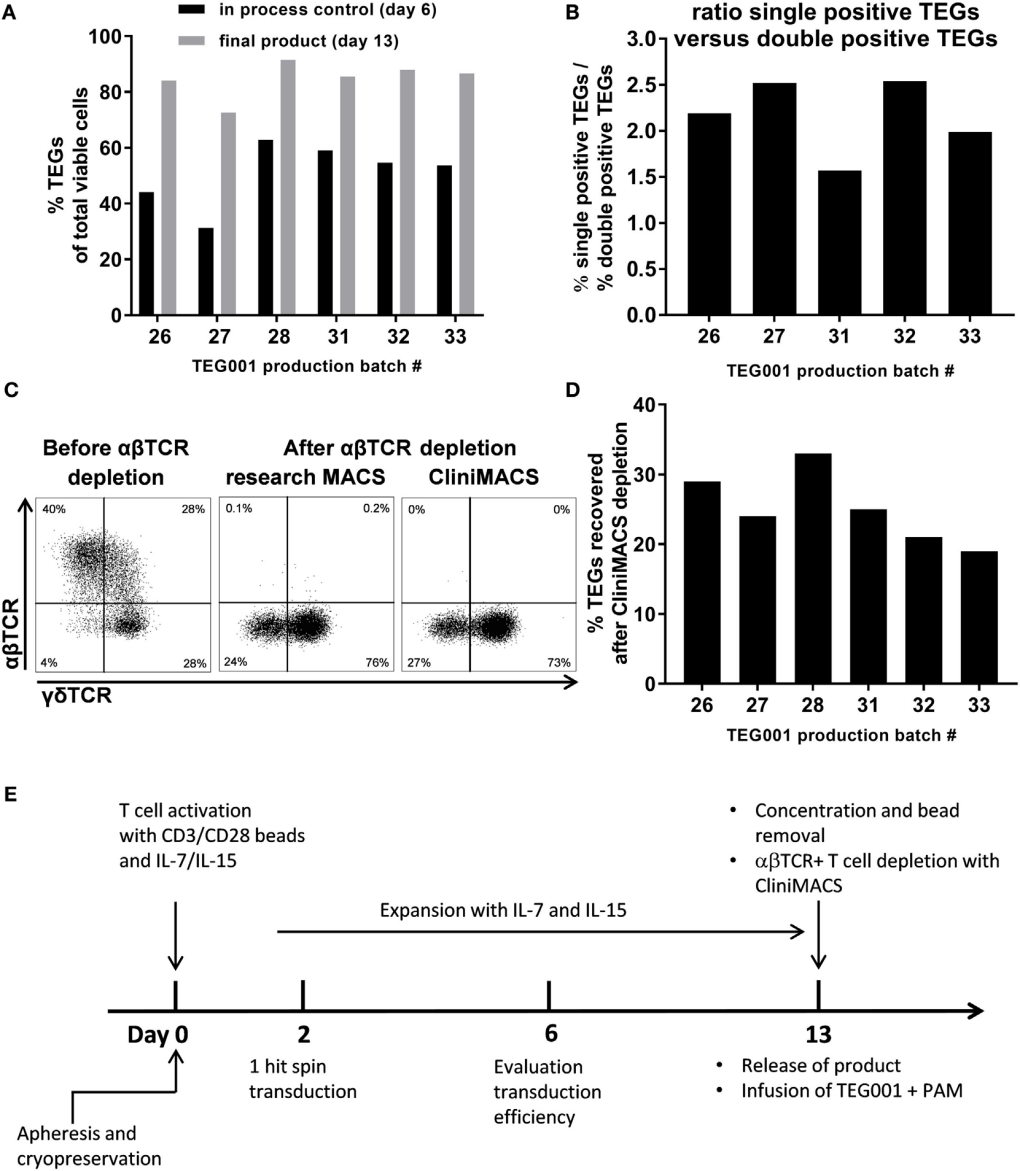
Successful enrichment of TEGs by CliniMACS depletion. **(A)** Comparison of TEGs transduction efficiency during production (in-process control, day 6) and at the end of production (final product) of six different production batches. **(B)** After introduction of pMP71:γTCR-T2A-δTCR and expansion of the T cells both γδTCR+αβTCR− T cells as γδTCR+αβTCR+ T cells are present. **(C)** During one of the research scale production batches, the cells at Day 13 were split and the non-transduced T cells were depleted using the research MACS or CliniMACS cell separation systems. **(D)** γδTCR+ cell recovery as percentage of the γδTCR+ cell input was measured after each αβTCR CliniMACS depletion. **(E)** Overview of the Good Manufacturing Practice TEG001 production process.

Non- and poorly-engineered cell fractions are usually present in genetically engineered T cell products and associate with little or no activity as shown also for TEG001 (Figures 4A,B). In addition, such cell fractions could even be harmful as they might harbor unwanted specificities. To avoid this potential drawback, a procedure for the depletion of non- and poorly-engineered T cells was developed by taking advantage of the observation that upon introduction of a γδTCR, the endogenous αβTCR expression is substantially decreased or even absent (2, 5). First, we defined “in process controls.” During six large-scale proof runs, the percentage of γδTCR-positive cells was, therefore, assessed at day 6 (in process control), before and after the CliniMACS depletion procedure. Between 31 and 63% of all cells were positive for γδTCR at day 6, and 73–92% at day 13 after CliniMACS depletion (Figure 5A). In addition, we assessed the robustness of the observation that introducing γδTCR substantially outcompetes endogenous αβTCRs during different GMP runs. The ratio of single positive to double positive TEGs ranged between 1.6 and 2.5 and was above 1.5 for all runs (Figure 5B). Then, we aimed to assess whether a depletion of non- and poorly-engineered TEGs could not only be performed with research devices (8), but also with GMP-grade αβTCR beads on a CliniMACS device. Therefore, we compared side by side the research-scale and large-scale depletion of non- and poorly-engineered TEGs through αβTCR depletion on a CliniMACS. After the depletion procedure, we observed a comparable purity of single positive TEGs after both procedures (research-grade: 73% versus clinical-grade: 76%; Figure 5C), while the remaining αβTCR positive T cell fraction was very low for both research-grade and GMP-grade (0.3 and 0.0%, respectively). The αβTCR negative γδTCR negative populations present in both research-grade and GMP-grade depleted products (24 and 27%, respectively) mainly consisted of NK cells (data not shown). This double negative population was present at the end of all manufacturing runs (*n* = 6, range 8–27%), and was donor and batch dependent. The recovery after CliniMACS αβTCR depletion, indicated as percentage of γδTCR + T cell output of the respective input, varied between 19 and 33% (*n* = 6, Figure 5D). This procedure allowed us to produce TEGs in numbers up to 2 × 10^9^ cells and is, therefore, sufficient to deliver dosages needed for the planned clinical study. The complete manufacturing schedule is depicted in Figure 5E.

### Immunological Phenotype of TEG001 Drug Substance

**Figure 6:**
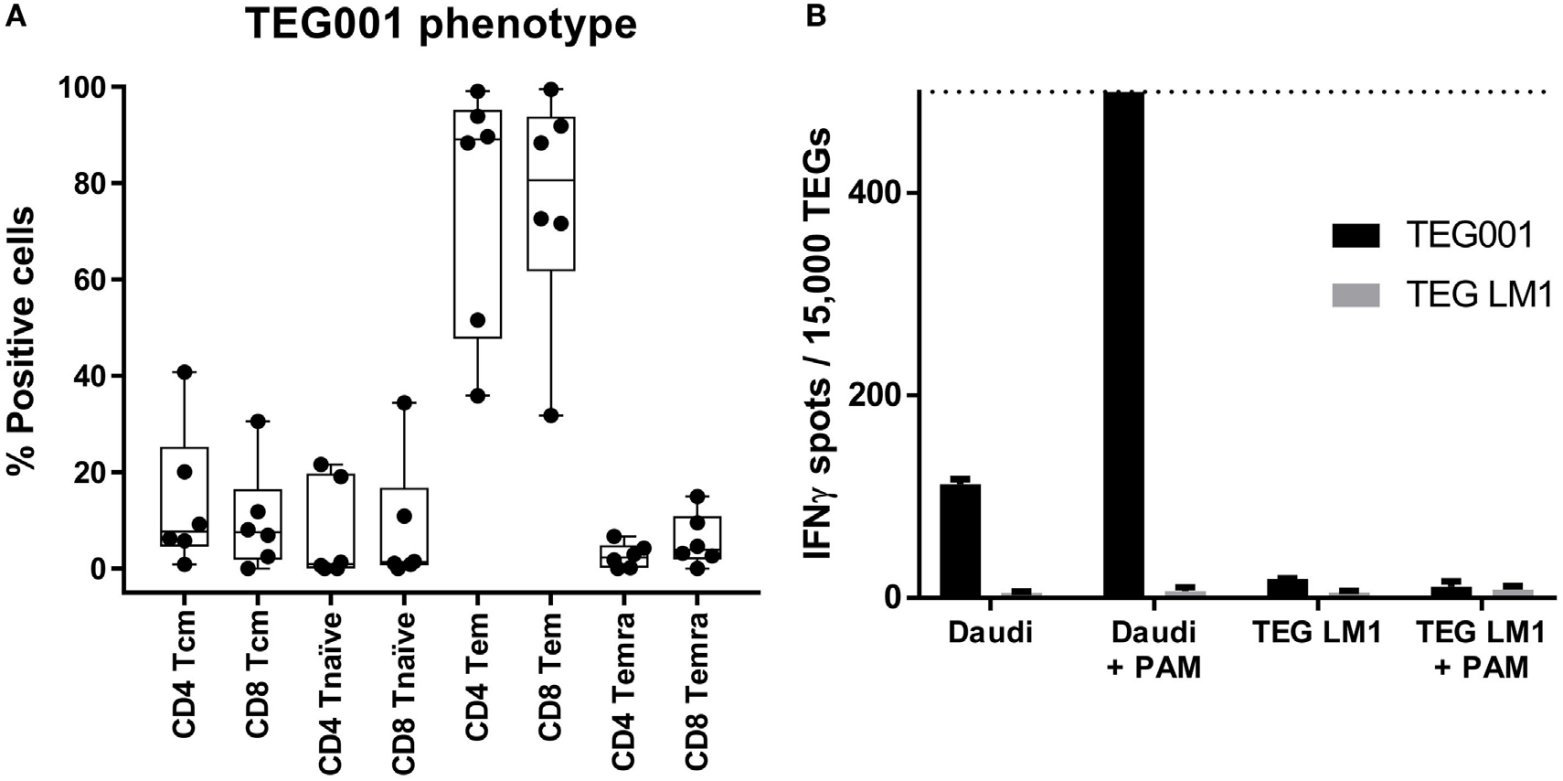
TEGs have a predominant effector-memory/effector phenotype. **(A)** The phenotype of TEGs from four different donors was determined by measuring CD45RO in combination with CD27 expression on day 13, after the CliniMACS depletion. CD45RO+/CD27+ is considered as *T*_cm_, CD45RO−/CD27+ as *T*_n_, CD45RO+/CD27− as *T*_em_, and CD45RO−/CD27− as *T*_emra_ (21). **(B)** TEGs were produced according to the described procedure after which they were stored at 2–8°C. After 20 h, the TEGs were coincubated with Daudi in the absence and presence of pamidronate (PAM) as a positive target, or TEG LM1 in the absence and presence of PAM as a negative target. TEG LM1 as effector served as the negative control. The maximum assay sensitivity was set at 500 spots (dashed line).

Next, we characterized the immunological phenotype of the drug substance TEG001, as the *in vivo* proliferation capacity and function of genetically modified cell therapy products is not only determined by the introduced receptor but also by the differentiation phenotype of the individual T cells. The differentiation phenotype of TEGs was determined by measuring the expression of CD27 and CD45RO (21) of five pre-GMP production runs. The major subset of TEGs was, after a 2-week expansion period, T cells with an effector (T_eff_) and effector memory (T_em_) phenotype (Figure 6A). In addition, all products contained engineered central memory T cells (T_cm_), an immune subset enabling potential long-term persistence of TEG *in vivo* (22).

### Release Specifications of TEG001 Drug Substance and TEG001 Drug Product, Batch Analyses, and Stability

Release specifications are an essential component of quality assurance and protect the patient from receiving a suboptimal cellular product. Based on our small-scale runs, we defined, therefore, product release specifications. To avoid overly stringent product definition criteria, which could result in the discarding of a product for clinical use, we defined release criteria for the TEG001 drug substance as ≥50% γδTCR positive T cells, ≥70% viability, and ≤10% αβTCR positive T cells in addition to standard microbiological release criteria (Table 1). All six large-scale runs met these release criteria as indicated in Table 2. In addition, after formulation of the drug product, stability of TEG001 drug product was assessed. This is essential for clinical practice as products are frequently administered for logistical reasons within 1 day after production is complete. Therefore, a fraction of TEG001 drug product was stored for 16 and 20 h at 4°C and tested for viability over time. All three tested batches remained stable over the tested time period (Table 3). Next, we tested whether GMP-grade TEG001 drug product is functional after storage for 20 h at 4°C, by co-incubation of TEG001 and TEG-LM1 (mock control) with the reference target cell line Daudi, in the presence of pamidronate (2, 8). TEG001 was effective in recognizing Daudi, while there was no recognition by mock TEGs when assessed by IFNγ ELISPOT (Figure 6B).

**Table 1:** Release specifications TEG001 drug substance.

Parameter	Method	Acceptance criteria
Identity		
- TEG001	Flow cytometry	Identity confirmed
Purity		
- % γδTCR-positive T cells	Flow cytometry	≥50%
- Viability	Manual cell count	≥70%
Impurities		
- % αβTCR-positive T cells^a^	Flow cytometry	≤10%
Microbiology		
- Sterility	Ph.Eur	Negative
- Mycoplasma	Ph.Eur	Negative
- Endotoxins	Ph.Eur	<2.0 IU/ml

*The TEG001 release specifications as defined in the investigational medicinal product dossier*.

*^a^Only applicable for patients who previously received an allogeneic hematopoietic stem cell transplantation*.

**Table 2:** TEG001 drug substance batch analysis data of large scale runs.

Parameter	Acceptance criteria	Run					
		26	27	28	31	32	33
Identity							
- TEG001	Identity confirmed	Pass	Pass	Pass	Pass	Pass	Pass

Purity							
- % γδTCR-positive T cells	≥50%	84%	73%	92%	86%	88%	87%
- Viability	≥70%	99%	98%	99%	97%	100%	100%

Impurities							
- % αβTCR-positive T cells^a^	≤10%	0.0%	0.0%	0.2%	0.5%	0.4%	0.1%

Microbiology							
- Sterility	Negative	ND	ND	Negative	Negative	ND	ND
- Mycoplasma	Negative	ND	ND	Negative	Negative	ND	ND
- Endotoxins	<2.0 IU/ml	ND	ND	Pass	Pass	ND	ND

*TEG001 was produced using our Good Manufacturing Practice large scale production protocol in multiple large scale manufacturing runs. TEG001 was formulated after which the identity, purity, and viability was assessed using trypan blue exclusion and flow cytometry*.

*^a^Only applicable for patients who previously received an allogeneic hematopoietic stem cell transplantation*.

**Table 3:** Stability data of TEG001 cell suspension for infusion stored at 2–8°C.

Time point	Parameter	Run (content TEG001 cells per 100 ml drug product)						
		31 69 × 10^6^	31 12 × 10^7^	32 71 × 10^6^	32 14 × 10^7^	32 26 × 10^7^	33 73 × 10^7^	33 31 × 10^7^
Formulation	Viability	97%	97%	100%	100%	100%	100%	99%
T = 0 h	Viable cell number recovery	100%	100%	100%	100%	100%	100%	100%

Storage	Viability	96%	97%	99%	100%	99%	100%	100%
T = 16 h	Viable cell number recovery	93%	94%	99%	94%	90%	100%	97%

Storage	Viability	94%	94%	100%	100%	99%	100%	100%
T = 20 h	Viable cell number recovery	84%	94%	99%	94%	90%	97%	97%

*TEG001 was produced using our Good Manufacturing Practice large scale production protocol in multiple large scale manufacturing runs. TEG001 was formulated in NaCl 0.9% for infusion with 4% HA at different cellular concentrations, to study the effect of TEGs density on cell viability and viable cell recovery at T = 0 and after storage at 2–8°C for 16 and 20 h*.

## Discussion

We have developed a robust GMP-grade TEG production protocol, which not only includes a conventional transduction and expansion step but also a very stringent CliniMACS enrichment procedure to guarantee high purity of the drug substance. This purification procedure can be used for any engineered immune cell product, which associates with a reduced expression of the αβTCR, like CAR-T introduced in the αTCR locus (23, 24). By utilizing this protocol, we have been able to produce and enrich TEGs in numbers, which are sufficient to reach the highest dose level of our upcoming phase I trial NTR6541. Furthermore, we have shown that γδTCR expression can be used as potency assay for TEG001, and that the TEG001 drug product is stable for at least 20 h at 4°, which allows for provisional release and transportation to the location of the infusion.

In current manufacturing processes of CAR-T cells, purification steps are often not included. As a consequence, final products currently infused into patients harbor only between 15 and 55% of engineered immune cells (18, 25, 26). The lack of purity can become a major clinical obstacle, in particular, when engineering T cells from patients who relapse after allogeneic stem cell transplantation. Re-infusion of CAR-T cells in patients after allogenic stem cell transplantation has been reported to associate with incidences of acute and chronic graft versus host disease (GvHD), up to 10% (27). GvHD after infusion of CAR-T cells is most likely a consequence of endogenous αβTCRs still expressed at physiological levels in CAR-T cells, as well as the presence of non-engineered immune cells within the product. With our TEG concept, we provide comprehensive solutions to these problems. First, as suggested from our previous data, not only in the research environment (8) but also with our presented GMP manufacturing process, endogenous αβTCRs are substantially downregulated in TEGs. Reduced expression of endogenous αβTCRs is most likely due to the efficient competition of the introduced γδTCR against endogenous αβTCR for CD3 components of a T cell. Second, the additional GMP enrichment procedure utilizing the CliniMACS system achieves purity of TEGs, which can exceed 90%. The removal of non- and poorly-engineered cells from the final drug substance has another advantage, in addition to the reduced risk of GvHD and a more potent product due to enrichment of γδTCR-positive cells; the improved competition for homeostatic cytokines (28, 29). Other strategies for the refinement of engineered immune cells have been developed recently. However, alternative purification strategies frequently depend on the introduction of an additional truncated protein such as CD19 or epidermal growth factor receptor, which are normally absent in the T cell lineage (30). Using a transgene cassette with an additional sequence for selection purposes can lead to lower transduction efficiencies and reduced expression of the introduced immune receptor or alternative homing (J. Kuball, unpublished observation) and associates frequently with unwanted T cell activation and immunogenicity (31). To the best of our knowledge, we have developed the very first GMP manufacturing procedure in which αβTCR depletion is used as a purification method, thereby delivering untouched clinical-grade engineered immune cells. Despite an efficient elimination of non- and poorly-engineered αβT cells, our procedure also enriches for NK cells. An additional purification step before T cell engineering might, therefore, be intriguing for the next generation of TEG-manufacturing, such as αβTCR+ or CD3+ cell selection before T cell activation (32), as proposed by others.

Advanced therapy medicinal products (ATMPs), such as TEGs, are individualized and complex biological products that require careful consideration of their nature in order to define adequate “in process” and “release” tests. ATMPs are also frequently freshly prepared and directly infused into patients after production, limiting the possibilities for extensive safety and release testing for an individual product. Despite the limited possibilities, regulatory authorities oblige a potency assay before batch release. Potency is defined as “the specific ability or capacity of the product, as indicated by appropriate laboratory tests or by adequately controlled clinical data obtained through the administration of the product in the manner intended, to effect a given result” (33). Potency must be measured in a robust and biologically relevant way, which reflects the mechanism of action. Thus, defining valid potency assays can be a major challenge for ATMPs. For CAR019, expression of the introduced receptor as a potency assay has been proposed by vendors and accepted by the FDA (17, 34). We provide now evidence that for TEGs, γδTCR expression levels are an adequate potency assay. However, expression levels of receptors are rather simplified and surrogate methods to assess for activity will not predict efficacy *in vivo*. Therefore, alternative methods are needed. High-throughput characterization of TEGs on single cell levels could be interesting alternatives, as previously also reported for CAR-T (35).

In conclusion, we have developed a GMP-grade manufacturing strategy for TEGs incorporating an αβTCR depletion to obtain a final product substantially enriched for TEGs. The described process can also be valuable for any CAR-T product interfering with endogenous αβTCR expression. We also defined release and potency criteria acceptable for competent authorities. TEG001 will be used for an upcoming phase I dose escalation clinical trial registered as NTR6541. This trial aims to investigate the safety and tolerability of TEG001 in patients with relapsed/refractory acute myeloid leukemia, high-risk myelodysplastic syndrome, and relapsed/refractory multiple myeloma.

## Author Contributions

JK and TS designed experiments. GK, TS, SH, RD, KJ, and JS performed the experiments, AM reviewed data and submitted documents to authorities; GK, TS, ZS, and JK wrote the manuscript; all authors agreed on the final manuscript.

## Conflict of Interest Statement

GK, ZS, and JK are inventors on different patents with γδTCR sequences, recognition mechanisms, and isolation strategies. JK is CSO and shareholder of Gadeta (www.gadeta.nl). The remaining authors declare that the research was conducted in the absence of any commercial or financial relationships that could be construed as a potential conflict of interest. The reviewer JA and handling Editor declared their shared affiliation.

## References

[1] Chabannon C Kuball J McGrath E Bader P Dufour C Lankester A CAR-T cells: the narrow path between hope and bankruptcy? Bone Marrow Transplant (2017) 52(12):1588–9. 10.1038/bmt.2017.241 29209061

[2] Grunder C van Dorp S Hol S Drent E Straetemans T Heijhuurs S gamma9 and delta2CDR3 domains regulate functional avidity of T cells harboring gamma9delta2TCRs. Blood (2012) 120(26):5153–62. 10.1182/blood-2012-05-432427 23018643

[3] Sebestyen Z Scheper W Vyborova A Gu S Rychnavska Z Schiffler M RhoB mediates phosphoantigen recognition by Vgamma9Vdelta2 T cell receptor. Cell Rep (2016) 15(9):1973–85. 10.1016/j.celrep.2016.04.081 27210746 PMC5035041

[4] Gu S Sachleben JR Boughter CT Nawrocka WI Borowska MT Tarrasch JT Phosphoantigen-induced conformational change of butyrophilin 3A1 (BTN3A1) and its implication on Vgamma9Vdelta2 T cell activation. Proc Natl Acad Sci U S A (2017) 114(35):E7311–20. 10.1073/pnas.1707547114 28807997 PMC5584448

[5] Marcu-Malina V Heijhuurs S van Buuren M Hartkamp L Strand S Sebestyen Z Redirecting alphabeta T cells against cancer cells by transfer of a broadly tumor-reactive gammadeltaT-cell receptor. Blood (2011) 118(1):50–9. 10.1182/blood-2010-12-325993 21566093

[6] Scheper W Grunder C Kuball J Multifunctional gammadelta T cells and their receptors for targeted anticancer immunotherapy. Oncoimmunology (2013) 2(5):e23974 10.4161/onci.23974 23762790 PMC3667896

[7] Scheper W Grunder C Straetemans T Sebestyen Z Kuball J. Hunting for clinical translation with innate-like immune cells and their receptors. Leukemia (2014) 28(6):1181–90. 10.1038/leu.2013.378 24345790

[8] Straetemans T Grunder C Heijhuurs S Hol S Slaper-Cortenbach I Bonig H Untouched GMP-ready purified engineered immune cells to treat cancer. Clin Cancer Res (2015) 21(17):3957–68. 10.1158/1078-0432.CCR-14-2860 25991821

[9] Braham MVJ Minnema MC Aarts T Sebestyen Z Straetemans T Vyborova A Cellular immunotherapy on primary multiple myeloma expanded in a 3D bone marrow niche model. Oncoimmunology (2018). 10.1080/2162402X.2018.1434465 PMC5980416 29872571

[10] Ghani K Cottin S Kamen A Caruso M. Generation of a high-titer packaging cell line for the production of retroviral vectors in suspension and serum-free media. Gene Ther (2007) 14(24):1705–11. 10.1038/sj.gt.3303039 17928873

[11] Ghani K Wang X de Campos-Lima PO Olszewska M Kamen A Riviere I Efficient human hematopoietic cell transduction using RD114- and GALV-pseudotyped retroviral vectors produced in suspension and serum-free media. Hum Gene Ther (2009) 20(9):966–74. 10.1089/hum.2009.001 19453219 PMC2861952

[12] Levine BL Bernstein WB Connors M Craighead N Lindsten T Thompson CB Effects of CD28 costimulation on long-term proliferation of CD4+ T cells in the absence of exogenous feeder cells. J Immunol (1997) 159(12):5921–30. 9550389

[13] Brentjens RJ Riviere I Park JH Davila ML Wang X Stefanski J Safety and persistence of adoptively transferred autologous CD19-targeted T cells in patients with relapsed or chemotherapy refractory B-cell leukemias. Blood (2011) 118(18):4817–28. 10.1182/blood-2011-04-348540 21849486 PMC3208293

[14] Gomez-Eerland R Nuijen B Heemskerk B van Rooij N van den Berg JH Beijnen JH Manufacture of gene-modified human T-cells with a memory stem/central memory phenotype. Hum Gene Ther Methods (2014) 25(5):277–87. 10.1089/hgtb.2014.004 25143008 PMC4208561

[15] Kalos M Levine BL Porter DL Katz S Grupp SA Bagg A T cells with chimeric antigen receptors have potent antitumor effects and can establish memory in patients with advanced leukemia. Sci Transl Med (2011) 3(95):95ra73. 10.1126/scitranslmed.3002842 21832238 PMC3393096

[16] Porter DL Levine BL Kalos M Bagg A June CH. Chimeric antigen receptor-modified T cells in chronic lymphoid leukemia. N Engl J Med (2011) 365(8):725–33. 10.1056/NEJMoa1103849 21830940 PMC3387277

[17] Levine BL Miskin J Wonnacott K Keir C Global manufacturing of CAR T cell therapy. Mol Ther Methods Clin Dev (2017) 4:92–101. 10.1016/j.omtm.2016.12.006 28344995 PMC5363291

[18] Lamers CH Willemsen RA Luider BA Debets R Bolhuis RL. Protocol for gene transduction and expansion of human T lymphocytes for clinical immunogene therapy of cancer. Cancer Gene Ther (2002) 9(7):613–23. 10.1038/sj.cgt.7700477 12082462

[19] Voss RH Kuball J Theobald M. Designing TCR for cancer immunotherapy. Methods Mol Med (2005) 109:229–56. 10.1385/1-59259-862-5:229 15585925

[20] Gaschet J Lim A Liem L Vivien R Hallet MM Harousseau JL Acute graft versus host disease due to T lymphocytes recognizing a single HLA-DPB1*0501 mismatch. J Clin Invest (1996) 98(1):100–7. 10.1172/JCI118753 8690780 PMC507405

[21] Klebanoff CA Gattinoni L Restifo NP. CD8+ T-cell memory in tumor immunology and immunotherapy. Immunol Rev (2006) 211:214–24. 10.1111/j.0105-2896.2006.00391.x 16824130 PMC1501075

[22] Sommermeyer D Hudecek M Kosasih PL Gogishvili T Maloney DG Turtle CJ Chimeric antigen receptor-modified T cells derived from defined CD8+ and CD4+ subsets confer superior antitumor reactivity in vivo. Leukemia (2016) 30(2):492–500. 10.1038/leu.2015.247 26369987 PMC4746098

[23] MacLeod DT Antony J Martin AJ Moser RJ Hekele A Wetzel KJ Integration of a CD19 CAR into the TCR alpha chain locus streamlines production of allogeneic gene-edited CAR T cells. Mol Ther (2017) 25(4):949–61. 10.1016/j.ymthe.2017.02.005 28237835 PMC5383629

[24] Legut M Dolton G Mian AA Ottmann O Sewell A CRISPR-mediated TCR replacement generates superior anticancer transgenic T-cells. Blood (2018) 131(3):311–22. 10.1182/blood-2017-05-787598 29122757 PMC5774207

[25] Lock D Mockel-Tenbrinck N Drechsel K Barth C Mauer D Schaser T Automated manufacturing of potent CD20-directed chimeric antigen receptor T cells for clinical use. Hum Gene Ther (2017) 28(10):914–25. 10.1089/hum.2017.111 28847167

[26] Hodi FS O’Day SJ McDermott DF Weber RW Sosman JA Haanen JB Improved survival with ipilimumab in patients with metastatic melanoma. N Engl J Med (2010) 363(8):711–23. 10.1056/NEJMoa1003466 20525992 PMC3549297

[27] Smith M Zakrzewski J James S Sadelain M Posttransplant chimeric antigen receptor therapy. Blood (2018) 131(10):1045–52. 10.1182/blood-2017-08-752121 29358181 PMC5865610

[28] Abad JD Wrzensinski C Overwijk W De Witte MA Jorritsma A Hsu C T-cell receptor gene therapy of established tumors in a murine melanoma model. J Immunother (2008) 31(1):1–6. 10.1097/CJI.0b013e31815c193f 18157006 PMC2235937

[29] de Witte MA Jorritsma A Kaiser A van den Boom MD Dokter M Bendle GM Requirements for effective antitumor responses of TCR transduced T cells. J Immunol (2008) 181(7):5128–36. 10.4049/jimmunol.181.7.5128 18802117

[30] Gardner RA Finney O Annesley C Brakke H Summers C Leger K Intent-to-treat leukemia remission by CD19 CAR T cells of defined formulation and dose in children and young adults. Blood (2017) 129(25):3322–31. 10.1182/blood-2017-02-769208 28408462 PMC5482103

[31] Traversari C Marktel S Magnani Z Mangia P Russo V Ciceri F The potential immunogenicity of the TK suicide gene does not prevent full clinical benefit associated with the use of TK-transduced donor lymphocytes in HSCT for hematologic malignancies. Blood (2007) 109(11):4708–15. 10.1182/blood-2006-04-015230 17327417

[32] Neurauter AA Bonyhadi M Lien E Nokleby L Ruud E Camacho S Cell isolation and expansion using Dynabeads. Adv Biochem Eng Biotechnol (2007) 106:41–73. 10.1007/10_2007_072 17680228

[33] FDA. 21 CFR 600.3(s). (2017). Available from: https://www.gpo.gov/fdsys/pkg/CFR-2011-title21-vol7/pdf/CFR-2011-title21-vol7-sec600-3.pdf (Accessed: January 25, 2018).

[34] FDA. BLA 125646 – Tisagenlecleucel. (2017). Available from: https://www.fda.gov/downloads/AdvisoryCommittees/CommitteesMeetingMaterials/Drugs/OncologicDrugsAdvisoryCommittee/UCM566166.pdf (Accessed: January 25, 2018).

[35] Xue Q Bettini E Paczkowski P Ng C Kaiser A McConnell T Single-cell multiplexed cytokine profiling of CD19 CAR-T cells reveals a diverse landscape of polyfunctional antigen-specific response. J Immunother Cancer (2017) 5(1):85. 10.1186/s40425-017-0293-7 29157295 PMC5697351

